# Effect of Neem (*Azadirachta indica* L.) on Lipid Oxidation in Raw Chilled Beef Patties

**DOI:** 10.3390/antiox8080305

**Published:** 2019-08-14

**Authors:** Manel Ouerfelli, Juliana Villasante, Leila Bettaieb Ben Kaâb, MaríaPilar Almajano

**Affiliations:** 1 Research Unit “Nutrition et Métabolisme Azotés et Protéines de Stress” (UR/ES-13-29), Biology Department, Faculty of Sciences of Tunis (FST), University of Tunis El-Manar (UTM), University Campus of Tunis El-Manar, 2092 Tunis, Tunisia; 2 Chemical Engineering Department (DEQ), School of Industrial Engineering of Barcelona (ETSEIB), Universitat Politècnica de Catalunya (UPC), Av, Diagonal 647, 08028 Barcelona, Spain

**Keywords:** *Azadirachta indica*, antimicrobial activity, antioxidants, lipid oxidation, beef meat

## Abstract

The aim of this study was to determine the total polyphenol content, radical scavenging and antimicrobial activities of *Azadirachta indica* (*A. indica*) and to evaluate their effect on shelf-life stability of raw beef patties during refrigerated storage at 4 ± 1 °C. During 11 days of storage, the antioxidant effect of *A. indica* on ground beef meat was investigated by the determination of lipid oxidation, pH, anti-radical activity, color, hexanal content, and microbial growth. The results obtained showed that fresh *A. indica* leaves and synthetic conservative behaved in the same way and retarded the lipid oxidation of chilled beef patties while increasing their pH (5.40 and 5.45, respectively). It can also be said that *A. indica* limited the loss of color, reduced the metmyoglobin formation (36.70%) and had a significant effect on bacterial growth and hexanal content. In addition, the results obtained through anti-radical and antimicrobial properties showed proportional values of total polyphenol content and radical scavenging activity of leaf extracts as they showed their antimicrobial effect against some bacteria such as *Staphylococcus aureus* and *Micrococcus luteus*, among others. These results support the involvement of *A. indica* in the food industry as a natural antioxidant that could replace synthetic ones.

## 1. Introduction

For decades, red meat from beef, sheep and pork has played an important role in the human diet [1] due to its high quality proteins, minerals, vitamins and many other essential nutrients indispensable for human health [2]. However, meat and meat products are susceptible to two main factors that lead to their deterioration, which are microbial growth and rapid lipid oxidation caused by the high concentrations of moisture, unsaturated lipids, hemo-pigments and other different oxidizing agents present in the muscle tissues [3]. 

Microbial growth and rapid lipid oxidation are generally accompanied by the formation of toxic compounds, texture deterioration, color and nutrient loss, accumulation of harmful compounds and shelf-life lessening, which decrease the nutritional quality of the meat products [4]. 

Therefore, different methods were developed to preserve meat and meat products from deterioration [5], like adding exogenous antioxidants considered beneficial for the meat quality. For instance, synthetic antioxidants such as the butylated hydroxytoluene (BHT) and butylated hydroxyanisole (BHA) were widely added in meat products to prevent and reduce lipid oxidation [6]. However, their application in meat products, has recently being questioned, mainly by consumers, due to their possible side effects [5,7]. For this reason, consumers tend to prefer meat products that contain the minimum quantities of synthetic antioxidants and prefer products with natural preservatives from plants with high antioxidant and antibacterial properties to reduce the risk of lipid oxidation in the meat and at the same time to ensure it is a healthy product for the consumer [8].

Several studies demonstrated the efficiency of plant materials rich in phenolic compounds as good natural antioxidants in meat and meat products. As examples, pomegranate peel [9], leaf extracts from *Rumex tingitamus* [10], Lucerne (alfalfa) [11], *Urtica dioica* and *Hibiscus sabdariffa* [12] can be cited. Moreover, plants represent good opportunities to control microorganisms in food as an alternative to synthetic preservatives [13]. For instance, *Azadirachta indica* A. Juss., commonly denominated as the Neem tree, which belongs to the Meliaceae family, is one of the most useful plants in traditional medicine in the Indian culture thanks to its various therapeutic benefits [14,15]. Neem is considered one of the most important plants worldwide, due to its diverse therapeutic applications and the variety of bioactive constituents present in its different parts [16]. The documented medicinal virtues of the Neem showed that its different parts (leaves, flowers, seeds, fruits, roots and bark) have been used to treat several human ailments such as inflammation, diarrhea, bacterial infection, constipation and even cancer [17].

Several reports suggested the importance of the Neem as an edible plant rich in bioactive molecules and pharmacological properties [18]. However, despite the diverse investigations done to demonstrate the biological and therapeutic benefits of Neem’s different parts, its effect on meat products remains unclear given the poorness of research regarding the use of Neem. For these reasons, the main target of this study is to determine phenolic compounds and antimicrobial activities of *A. indica* leaf extracts and evaluate the antioxidant property of its dry leaves on lipid oxidation and shelf life of raw beef patties during refrigerated storage at 4 ± 1 °C.

## 2. Materials and Methods

### 2.1. Natural Products

The leaves of *A. indica* were collected from Punjab in the north of India; while *C. baccatum* fruits were purchased from a local market (Mercadona, Barcelona, Spain).

### 2.2. Microbial Strains

Six different microbial strains from the collection ATCC were obtained from the Universitat de Barcelona (UB). *Staphylococcus aureus* (*S. aureus*, ATCC 25423), *Micrococcus luteus* (*M. luteus*, ATCC 4698), *Listeria* (ATCC 15313) and *Bacillus cereus* (*B. cereus*, ATCC 11778) are Gram-positive (Gram+) bacteria, while *Escherichia coli* (*E. coli*, ATCC 25022) and *Salmonella paratyphi A* (*S. paratyphi*, ATCC 9150) are Gram-negative (Gram−).

### 2.3. Meat

Fresh ground beef meat was purchased from “Carns Blai” butchery (Barcelona, Spain) and brought to the laboratory under refrigeration (4 ± 1 °C).

### 2.4. Chemicals and Products

Ethanol (EtOH), methanol (MeOH), gallic acid (GA), trolox, 2-thiobarbituric acids (TBA), 2,2-diphenyl-1-picrylhydrazyl (DPPH), hexanal and peptone water were purchased from Sigma-Aldrich Química S.A. (Madrid, Spain). Acetone (Ac), Folin–Ciocalteu reagent and phosphate buffered saline (PBS) were acquired from Panreac Química S.L.U (Barcelona, Spain). Miller Hinton agar, tryptone glucose yeast agar and Penicillin-Streptomycin (Pen-Strep) were bought from Thermo-Fisher Scientific (Barcelona, Spain).

The synthetic preservative used as a positive control in the determination of the antioxidant effect of powdered *A. indica* dry leaves on beef meat quality is “Conservative CAMPA N°3 (A), code 403600”, elaborated by “La Campana” for burger meat and composed of: dextrose, preservatives: (sulfur dioxide 5.7%), E-224 (sulfite) and antioxidants (E-301 and E-331 iii). The synthetic preservative was purchased from la CAMPANA (Barcelona, Spain).

### 2.5. Instrumentation 

All absorbance analyses were performed on multimode micro-plate reader FLUOstar^®^ Omega (Ortenberg, Germany) equipped with five detection modes using an ultra-fast UV/Vis. 

### 2.6. Total Polyphenol Content and Radical Scavenging Activity of A. indica and C. baccatum Extracts

#### 2.6.1. Extracts Preparation

Dry leaves of *A. indica* and *C. baccatum* were ground with liquid nitrogen until fine and homogeneous powders were obtained. The extraction was carried out according to the method of Slatnar et al. [19] with few adaptations. An amount of 1 g from each powdered sample was extracted with 10 mL of 50% aqueous EtOH (v/v) at 4 ± 1 °C under sonication for 1 h. Then, the extracts were centrifuged at 16,800× *g* for 10 min at 4 °C (Orto Alresa Mod. Consul, Ortoalresa, Ajalvir Madrid, Spain). The supernatants obtained were filtered through Whatman ﬁlters N°1 and lyophilized for 2 days (Unicryo MC2L, UniEquip Laborgerätebau & Vertr. GmbH, Martinsried, Munich, Germany). The final dry extracts obtained were dissolved in 5 mL of different solvents with increased polarity (80% EtOH, 80% MeOH and deionized water) then stored at 4 °C after being filtered through 0.45 µm pore filters to ensure their purity. All the extracts were used to determine their total polyphenols content and radical scavenging activity, following DPPH assay, and 80% MeOH samples were used to assess their antimicrobial activity.

For all parameters studied below, samples were analyzed in triplicate.

#### 2.6.2. Total Polyphenols Content (TPC)

TPC was determined following the Folin–Ciocalteu phenol reagent method as described by Villasante et al. [20]. The absorbance was measured at 765 nm and measurements were based on a calibration curve made with Gallic Acid (100–1700 µM, R^2^ = 0.992). The results are expressed as milligrams of gallic acid equivalent per gram of dry weight (mg GAE/g DW).

#### 2.6.3. Radical Scavenging Activity (RSA): DPPH Assay

The ability of *A. indica* and *C. baccatum* extracts to scavenge DPPH radicals was assessed following the method adapted by Maqsood and Benjakul [21]. The results are expressed as milli-mole of Trolox equivalents per gram of dry weight (mM TE/g of DW).

### 2.7. Antibacterial Activity of A. indica and C. baccatum Extracts, by Agar Disk-Diffusion Method (Inhibitory Zone Assay)

Inhibitory zone assay was assessed following the method of Balouiri et al. [22] using sterile disks (inner diameter 6 mm). The 80% aqueous MeOH extract of *A. indica* and *C. baccatum* were sterilized by filtration through 0.22 μm milli-pore filters. Mueller–Hinton agar was solidified in different culture dishes and each dish was inoculated with 100 μL of a different bacterial suspension. Then, sterile disks impregnated with 100 μL of each extract were put in each dish. Disks soaked in penicillin (100 μg/mL) were used as positive control, while disks soaked in 80% MeOH were used as negative control. All the culture plates were incubated at 35 ± 2 °C for 18 h. The antibacterial activity of the different samples was determined by measuring the diameter of inhibition zones against the tested microorganisms.

### 2.8. Antioxidant Effect of Powdered A. indica Dry Leaves on Beef Meat Quality

#### 2.8.1. Preparation and Storage Conditions of Raw Beef Patties

Three minced meat pieces of three different cuts taken from the round part of three different beef were bought fresh on three different days. The meat mixture was done by mincing the meat twice and passing it through a 4 mm diameter hole, so that the fat was homogeneously distributed. Each piece was well mixed with salt (1.5%, w/w) and divided into five different parts, one part was considered as a control and each one of the remaining four parts was mixed with a different compound to finally obtain five different beef meat parts: CTR (CTR, meat sample with no antioxidant), S.C (meat samples formulated with 0.7% (w/w) synthetic conservative), *C.B* (meat samples formulated with 0.7% (w/w) powdered *C. baccatum* fruits), *A.I* (meat sample formulated with 0.7% (w/w) *A. indica* dry powdered leaves) and *A.I* + *C.B* (meat samples formulated with 0.7% (w/w) fresh powdered *A. indica* leaves combined with 0.7% (w/w) powdered *C. baccatum* fruits). 

Various patties (3 to 4 g in weight, 4 cm in diameter and 0.5 cm in thickness) were subsequently formed using a round cutter, then placed in plastic trays after being covered with plastic films and kept in the refrigerator at 4 ± 1 °C. During 11 days of chilled storage, the lipid oxidation delay and metmyoglobin (MetMb) content were measured every 2 days while the quality of beef patties (pH and color variations) was monitored daily. The microbiological analysis, the anti-radical activity determined by the hydrophilic and lipophilic DPPH assays, and the determination of hexanal content were carried out every 5 days.

#### 2.8.2. Lipid Oxidation and pH Value Evolution

Evolution of lipid oxidation in raw beef patties was assessed by thiobarbituric acid reactive substance (TBARS) assay as described by Fan et al. [23]. The absorbance was measured at 531 nm and the results are expressed as milligrams of malondialdehyde (MDA) per kilogram of meat sample (mg MDA/kg meat sample).

The pH measurement of beef samples was determined using an Orion 3-Star pH Benchtop Meter (Thermo Fisher Scientific, Waltham, MA, USA).

#### 2.8.3. Antioxidant Capacity (AOC)

AOC of meat samples was determined by hydrophilic and lipophilic DPPH assays (H-DPPH and L-DPPH, respectively). As described by Gallego et al. [24], the first extract solvent was Milli-Q water (Simplicity^®^, C9210, Merck KGaA, Darmstadt, Germany) and the second one was composed of Ac, EtOH and Milli-Q water (5:4:1; v/v/v) to extract hydrophilic and lipophilic anti-radicals, respectively. The extracts obtained were used to perform DPPH assays as described previously. The results were expressed as micromole of Trolox equivalents per milliliter of extract (μmol TE/mL).

#### 2.8.4. Color Fading Measurement

Color fading of the different treated meat samples was measured in triplicate at three different locations on the surface of the raw beef patties while avoiding the fatty zones in order to obtain correct measurements [25]. Color stability of raw beef patties was evaluated using a reflectance colorimeter Minolta CR-400 (Konica Minolta, Tokyo, Japan) and was expressed against the scale of Lightness (L*), Redness (a*) and Yellowness (b*) in the CIELab color space system. 

#### 2.8.5. Metmyoglobin (MetMb) Reducing Activity

According to Aini et al. [26], an amount of 5 g from each beef meat sample was homogenized with 25 mL of PBS (0.04M, pH = 6.8) for 30 sec using Ultra Turrax (IKA, Model T18 Basic, Germany). The homogenized mixture was stored for 1 h at 4 °C and centrifuged at 1500× *g* for 20 min at 4 °C. The absorbance of the supernatant was read at 572, 565, 545 and 525 nm. The percentage of MetMb (%) was determined using the Krzywicki equation (1) described below:

MetMb (%) = [2.514 × (A_572_/A_525_) + 0.777 × (A_565_/A_525_) + 0.8 × (A_545_/A_525_) + 1.098] (1)

where A_572_ is absorbance at a wavelength of 572 nm, A_525_ is absorbance at a wavelength of 525 nm, A_565_ is absorbance at a wavelength of 565 nm and A_545_ is absorbance at a wavelength of 545 nm.

#### 2.8.6. Hexanal Content Determination by HS-GC/MS 

The different meat samples were prepared by mixing 500 mg of each treated beef sample with 1.5 mL of Milli-Q water in a headspace vial. Then, each vial was sealed air-tight with a PTFE septum. Hexanal was used as standard for the calibration at different concentrations ranging from 0.005 to 0.250 ppm. To determine the hexanal content, vials were incubated at 80 °C for 30 min. The analysis was performed using HS-GC/MS by injecting 1 mL of vapor phase through a syringe kept at 85 °C. Equipment used consisted of a Trace GC gas chromatograph with a Head Space Tri-plus auto-sampler coupled to a DSQII mass spectrometer (Thermo Fisher Scientific, Austin, Texas, USA) with TRB-624 (60 m × 0.32 mm × 1.8 mm) column, 1.8 mL/min helium flow. The injector temperature was 220 °C with split mode injection (split flow 20 mL/min). Temperature program was 60 °C held for 2 min and then raised to 220 °C at the rate of 8 °C /min (5 min). Interface temperature was 260 °C and ionization source temperature 230 °C [27]. Results are expressed in milligram of hexanal per gram of meat sample (mg hexanal/g meat sample).

#### 2.8.7. Microbial Analysis

The presence of colony-forming units was determined according to the method described by Hawashin et al. [28]. Shortly, 10 g of each beef patty were weighted into a stomacher bag (Stomacher^®^ Lab system, Seward) and homogenized with 100 mL of 0.1% sterile peptone water, then well mixed using a stomacher (Stomacher^®^ 80 Lab Blender, Galileo Equipos, Madrid, Spain) for 2 to 3 min. Different dilutions were prepared and 100 µL of each dilution were transferred to a standard agar coated plate (with Tryptone Glucose Yeast Agar, TGYA). The plates were incubated at 37 °C for 48 h. The microbial colonies were observed on the initial, fifth and last days of analysis. 

#### 2.8.8. Sensory Analysis

A panel composed of seven semi-trained judges, familiar with quality of meat and meat products, evaluated the cooked beef patties on day 1. The subjects were gender balanced (four females and three males) between the age of 20 and 28 years old. Each judge was instructed to taste each sample and grade them from 1 (least preferred) to 10 (most preferred) [29]. Control beef patties (without antioxidant) and beef patties formulated with synthetic conservative, *A. indica* leaves and *C. baccatum* fruits were cooked in a Hamburger Grill (Tristar, GR-2843, Barcelona, Spain) at full power for 5 min then coded and presented directly to the panelists. Water, apples and biscuits were provided to clean the palate after tasting each sample. Results were analyzed using the tables developed by Basker [30]. 

#### 2.8.9. Statistical Analysis

Data analysis was carried out in triplicate (*n* = 3) and standard deviations (SD) were calculated by the MINITAB software program (Version 17, München, Germany). Tukey’s test was used to calculate the significant differences (*p* < 0.05) between mean values.

## 3. Results and Discussion

### 3.1. Total Polyphenol Content (TPC) and Radical Scavenging Activity (RSA)

TPC and RSA of *A. indica* and *C. baccatum* extracts were determined and the results obtained are presented in Table 1.

Depending on the extraction solvent polarity, TPC results showed significant differences (*p* < 0.05) between samples. The leaves of *A. indica* extracted in 80% MeOH presented higher content of total polyphenols with 107.41 ± 0.03 mg GAE/g DW than extracts prepared in 80% EtOH and deionized water which contained lower TPC estimated at 47.47 ± 0.03 and 20.93 ± 0.08 mg GAE/g DW, respectively. TPC was also highest in *C. baccatum* fruits extracted in 80% MeOH with 53.91 ± 0.02 mg GAE/g DW followed by 80% EtOH extract with 34.78 ± 0.03 mg GAE/g DW and deionized water extract which contained the lowest content determined at 20.23 ± 0.01 mg GAE/g LP. Furthermore, the data obtained from the determination of *A. indica* leaf and *C. baccatum* fruit extract ability to scavenge DPPH radicals also presented significant differences (*p* < 0.05) between samples (Table 1). RSA was significantly higher (*p* < 0.05) in *A. indica* leaves and *C. baccatum* fruits extracted in 80% MeOH (0.72 ± 0.004 and 0.42 ± 0.001 mM TE/g LP, respectively) than 80% EtOH and deionized water extracts.

From the point of view of the samples investigated, *A. indica* leaves 80% MeOH, 80% EtOH and deionized water extracts presented, respectively, 27, 50 and 3% higher TPC and 22, 42 and 37% stronger RSA than *C. baccatum* extracts.

Variations in polyphenol contents and anti-radical activity were observed in different studies. For example, Ghimeray et al. [31] reported similar results for TPC expressed in tannic acid equivalents in *A. indica* leaves extracted in MeOH and water. However, RSA was higher in the present study. Datta et al. [32] reported lower TPC in *A. indica* leaves extracted with water than those obtained in this study. In addition, Sora et al. [33] made a comparative study of the phenolic content and antioxidant activity of *C. baccatum* ethanol extract and found TPC and RSA 5 and 7 times, respectively, higher than those found in the present study.

These variations in TPC could be explained by the type of extraction solvent used. MeOH and EtOH extracted polyphenols better in comparison with deionized water. This can be explained by the fact that the extraction yield of polyphenols is higher with solvents that have lower polarities than water [34]. The degradation of phenolic compounds by enzymes called polyphenol oxidases can also be an origin of the low TPC in the samples extracted with deionized water since these enzymes are active in water whereas they are inactive in alcoholic solutions [35]. This great variability found in literature values could also be related to the origin of each plant studied [20]. Moreover, the results collected by the TPC determination and the measurement of the anti-radical potential of *A. indica* and *C. baccatum* extracts to scavenge DPPH radicals revealed that the variation of the anti-radical activity observed in the different extracts of the two plants investigated depends on the TPC. In fact, the values obtained vary proportionally; the higher the polyphenols content, the stronger the antioxidant activity, which shows that the antioxidant activity of the various extracts may be due to their TPC. This agrees with the result found by Gallego et al. [36] and Kaviarasan et al. [37] who demonstrated that phenolic compounds are indeed responsible for the antioxidant activity of plant extracts [38].

### 3.2. Screening of Antibacterial Activity 

The results obtained (Table 2) showed that *A. indica* and *C. baccatum* 80% MeOH extracts had antimicrobial activity against the majority of bacterial strains tested. The best antimicrobial activity of *A. indica* extract was obtained in an *E. coli* strain with an inhibition zone estimated at 21 mm, followed by *S. aureus*, *M. luteus and S. paratyphi* strains with inhibition zones estimated at 19, 12 and 10 mm, respectively. However, *A. indica* extract had no activity on *Listeria* and *B. cereus* strains which had inhibition diameters of 15 and 10 mm, respectively, in the presence of penicillin. In addition, inhibition halos were observed in the presence of the *C. baccatum* extract. The strongest antimicrobial activity of *C. baccatum* extract was recorded in *S. aureus* strain with an inhibition zone estimated at 22 mm, followed by *M. luteus* and *Listeria* strains with inhibition zones estimated both at 10 mm. However, *C. baccatum* extract had no activity on *B. cereus*, *S. paratyphi* and *E. coli* strains which had diameters of 10, 30 and 27 mm, respectively, in the presence of penicillin.

The analysis of the results in Table 2 showed that 80% MeOH *A. indica* extract had antibacterial activity on both Gram-positive and Gram-negative bacteria, while *C. baccatum* extract had only an effect on Gram-positive bacteria. On one hand, this antibacterial activity is conferred by the presence of capsaicin and dihydrocapsaicin in extracts in addition to phenolic compounds whose antibacterial properties have been demonstrated in many researches [39,40]. On the other hand, the absence of antimicrobial activity in *A. indica* and *C. baccatum* against some bacterial strains could be explained by the fact that these strains developed resistance mechanisms. Many studies have shown that phenotypic variability may be a strategy put in place by certain microorganisms to resist certain compounds [41,42]. It is also possible that the solvent used during the extraction may not have been able to retain the desired molecules because of its polarity or concentration. Several investigations suggested that the antimicrobial activity of herbal extracts required high concentrations [43]. This probably suggests that the extract concentration used in the present study was lower than expected and this could explain the lack of activity.

In conclusion, different factors may be at the origin of the presence or absence of the extracts’ antibacterial activity. It all depends on the type of bacterial strain and its resistance, the polarity of the extraction solvent, the concentration of the extract and the composition and activity of the plant investigated.

### 3.3. Effect of Powdered A. indica Leaves on Beef Meat Quality During Refrigerated Storage

#### 3.3.1. Lipid Oxidation and pH Variation

**Figure 1:**
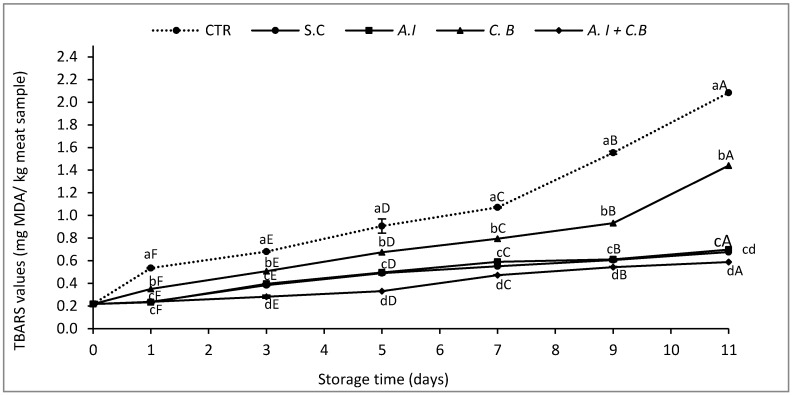
Thiobarbituric acid reactive substance (TBARS) values of raw beef patties during storage at 4 ± 1 °C. CTR (Control sample without antioxidant), S.C (treatment with 0.7% (w/w) synthetic conservative), *A.I* (treatment with 0.7% (w/w) powdered *A. indica* leaves), *C.B* (treatment with 0.7% (w/w) powdered *C. baccatum* fruits) and *A.I + C.B* (treatment with 0.7% (w/w) powdered *A. indica* leaves and 0.7% (w/w) powdered *C. baccatum* fruits). Results represent the mean of three replicates (*n* = 3) and are expressed as mean value ± SD; different letters in the same day indicate significant difference between samples at *p* < 0.05, different capital letters indicate significant difference between storage days at *p* < 0.05 for the same sample.

The direct incorporation of *A. indica* dry leaves in chilled ground raw beef meat has been carried out in order to evaluate their efficacy against the formation of malonaldehyde, aldehyde compounds, and ketones resulting from lipid oxidation. This evaluation was determined by the TBARS assay and the results obtained are illustrated in Figure 1.

The results showed that the secondary oxidation of beef samples significantly increased during refrigerated storage (*p* < 0.05). CTR beef sample had the highest TBARS value estimated at 2.08 mg MDA/kg meat compared with *A.I + C.B* (0.7%, w/w) which presented a synergistic antioxidant effect in raw beef patties and produced a combined inhibitory effect of MDA formation greater than the rest of formulated meat samples and reached a value of 0.59 mg MDA /kg meat by the end of the storage period. Secondary oxidation of *A.I*, S.C and *C.B* meat samples also increased progressively with storage time. The *A.I* meat sample showed an effective antioxidant effect against lipid degradation almost similar to the S.C sample and reached 0.68 and 0.70 mg MDA/kg meat, respectively, while *C.B* beef samples presented higher TBARS values estimated at 1.44 mg MDA/kg meat. TBARS values recorded in *A.I* and *A.I + C.B* beef patties were considered to be a good sign of their efficiency against lipid oxidation in beef patties since they didn’t exceed 1.5 mg MDA/kg meat. This value is considered as an indicator of lipid degradation in meat as reported by Martínez et al. [44].

**Figure 2:**
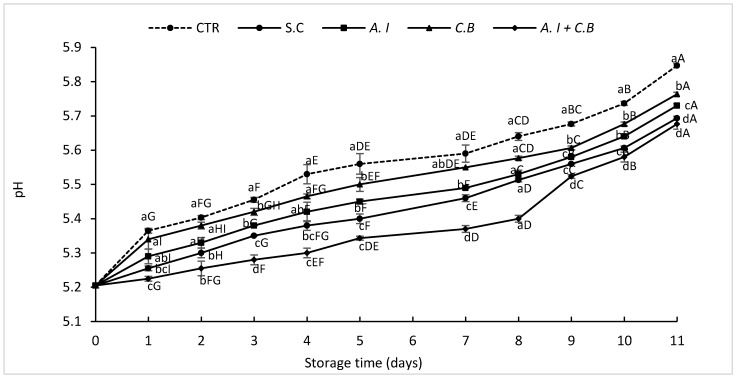
Evolution of pH values of raw beef patties during storage at 4 ± 1 °C. CTR (Control sample without antioxidant), S.C (treatment with 0.7% (w/w) synthetic conservative), *A.I* (treatment with 0.7% (w/w) powdered *A. indica* leaves), *C.B* (treatment with 0.7% (w/w) powdered *C. baccatum* fruits) and *A.I* + *C.B* (treatment with 0.7% (w/w) powdered *A. indica* leaves and 0.7% (w/w) powdered *C. baccatum* fruits). Results represent the mean of three replicates (*n* = 3) and are expressed as mean value ± SD; different letters in the same day indicate significant difference between samples at *p* < 0.05, different capital letters indicate significant difference between storage days at *p* < 0.05 for the same sample.

The pH of beef patties throughout chilled storage period was also measured to determine its correlation with TBARS values. The results obtained are presented in Figure 2.

At every storage day, the pH values were significantly different between meat samples (*p* < 0.05) and increased steadily over storage time. The highest pH values were recorded in the CTR meat sample which increased from 5.21 at day 1 to 5.85 at day 11 followed by the pH of *C.B* beef patties presenting pH values ranging from 5.21 to 5.76 at day 1 and day 11, respectively. The S.C, *A.I* and *A.I + C.B* kept their pH relatively low, especially *A.I + C.B* beef meat which presented the best pH values ranging from 5.21 in the initial day to 5.37 in the final day 11 of chilled storage.

Our findings about the positive impact of edible plants rich in phenolic compounds on the lipid oxidation process in meat are in agreement with other studies. Özer et al. [45] assessed the effects of quinoa flour on lipid oxidation in raw beef burger during long term frozen storage and found that the addition of quinoa significantly decreased TBARS values for raw burger compared to control group during storage. Abdelhakam et al. [46], also studied the quality characteristics of beef hamburgers enriched with red grape pomace powder during freezing storage and found similar results. The antioxidant effect of roasted coffees added to refrigerated ground pork over 21 days was determined by Hashimoto et al. [47] who found TBARS values in treated meat samples lower than those of control. Fan et al. [23] investigated the effects of *Portulaca oleracea* L. on lipid oxidation of pork meat during refrigerated storage and obtained results supporting the hypothesis that the addition of natural products enriched in polyphenols extends the shelf life of fresh meat and delays lipid oxidation.

The antioxidant effect of *A. indica* dry leaves and *C. baccatum* fruits on oxidative stability could possibly be associated with their wealth of phenolic compounds which present strong antioxidant activity allowing them to scavenge hydroperoxides, whose decomposition results in secondary oxidation products responsible for the deterioration of meat quality [48]. 

Moreover, the variation in pH values in beef meat during chilled storage can be influenced by different factors. Many studies have addressed the decrease in acidity on microorganisms and enzymes that degrade meat proteins and produce ammonia, amines and other toxic compounds. This conducts to high pH values. These compounds are formed rapidly when meat starts to decompose [49].

#### 3.3.2. AOC Assay 

**Figure 3:**
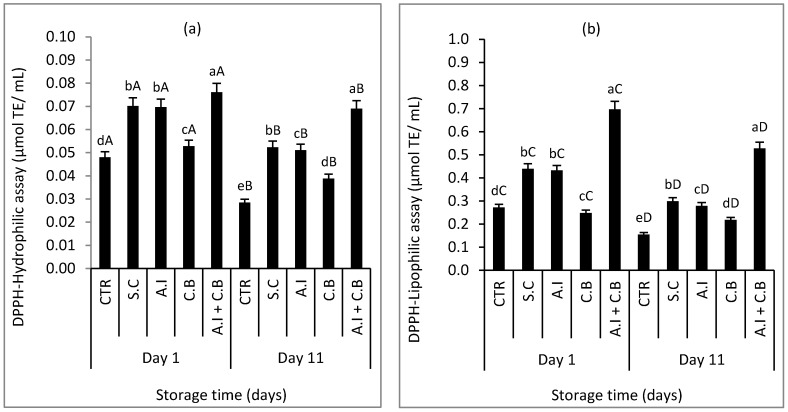
Antioxidant capacity (AOC) measurements by DPPH-Hydrophilic (**a**) and DPPH-Lipophilic (**b**) assays of raw beef patties during storage at 4 ± 1 °C. CTR (Control sample without antioxidant), S.C (treatment with 0.7% (w/w) synthetic conservative), *A.I* (treatment with 0.7% (w/w) powdered *A. indica* leaves), *C.B* (treatment with 0.7% (w/w) powdered *C. baccatum* fruits) and *A.I + C.B* (treatment with 0.7% (w/w) powdered *A. indica* leaves and 0.7% (w/w) powdered *C. baccatum* fruits). Results represent the mean of three replicates (*n* = 3) and are expressed as mean value ± SD; different letters in the same day indicate significant differences between samples at *p* < 0.05, different capital letters indicate significant differences between storage days at *p* < 0.05 for the same sample.

The results of the anti-radical capacity assay determined by the H-DPPH and L-DPPH assays in the initial and last day of refrigerated storage are summarized in Figure 3a,b, respectively.

The results obtained showed that AOC values determined by the H-DPPH assay (Figure 3a) are lower than those obtained by L-DPPH assay (Figure 3b). According to the H-DPPH assay, the result differences obtained are significant (*p* < 0.05) between samples on each day of analysis. The different samples exhibited stronger anti-radical activity at day 1 than day 11. The highest value was recorded in the meat sample formulated with *A.I + C.B* ranging from 0.08 to 0.07 μmol TE/mL, followed by the S.C *and A.I* beef samples which had almost the same anti-radical activity ranging from 0.07 to 0.05 μmol TE/mL and had no significant difference at day 1. CTR and *C.B* meat samples presented the lowest AOC values estimated at 0.05 and 0.03 μmol TE/mL at day 1 and 0.05 and 0.04 μmol TE/mL at day 11, respectively.

AOC values determined by L-DPPH assay (Figure 3b) also presented significant differences between samples (*p* < 0.05). CTR and *C.B* beef meat had the lowest anti-radical activities estimated at 0.27 and 0.25 μmol TE/mL in day 1 and 0.16 and 0.22 μmol TE/mL at day 11. The significantly highest values were recorded in *A.I + C.B* sample (0.70 and 0.53 μmol TE/mL at day 1 and 11, respectively), followed by S.C and *A.I* beef samples (with no significant difference at day 1) ranging from 0.44 to 0.30 μmol TE/mL at day 1 and 0.43 to 0.28 μmol TE/mL at day 11, respectively.

Lipophilic antioxidants, such as tocopherols and carotenoids, and hydrophilic antioxidants like ascorbic acid and the majority of phenolic compounds are two different groups of antioxidants which contribute to a high antioxidant capacity, protecting the meat products treated with natural antioxidants to be against oxidation [50]. Several studies demonstrated that these antioxidants can improve the nutrition value of meat. For instance, Gallego et al. [24] and Ouerfelli et al. [50] achieved similar results with differences in the values obtained and found that the *Caesalpinia decapetala* and *Anthyllis vulneraria,* respectively, can be good sources of natural antioxidants since they had higher antioxidant capacity determined with hydrophilic and lipophilic FRAP assays as they noticed that hydrophilic FRAP values are higher than lipophilic ones.

#### 3.3.3. Color Fading and MetMb Reducing Activity

Color changes measured on the surface of the different beef patties during 11 days of refrigerated storage are illustrated in Table 3.

The red color of meat is one of the most important factors that determines the purchase decision of consumers. The results presented in the Table 3 showed Lightness (L*) values of the different treated beef patties which decreased significantly (*p* < 0.05) over storage time.

The results showed also that the beef patties formulated with synthetic conservative had the highest Redness (a*) values ranging from 50.12 at day 1 to 37.21 at day 11, followed by the sample containing *A.I* + *C.B*, while CTR and *C.B* meat samples presented the lowest values during storage period, because it is meat that has a higher proportion of metmyoglobin, a color with a tendency to brown. On the other hand CTR and *C.B* meat samples presented the lowest values during storage period. *A.I* beef samples showed better red color values than the control samples despite the dark color that the powdered leaves attributed to the beef patties. 

In addition to Redness (a*), synthetic conservative enhanced the Yellowness (b*) of raw beef patties during storage period and presented lower values ranging from 15.25 at day 1 to 10.27 at day 11, compared to the Yellowness (b*) values of the CTR and *C. B* meat samples which decreased significantly (*p* < 0.05) during refrigerated storage and presented the lowest Yellowness (b*) values. 

**Figure 4:**
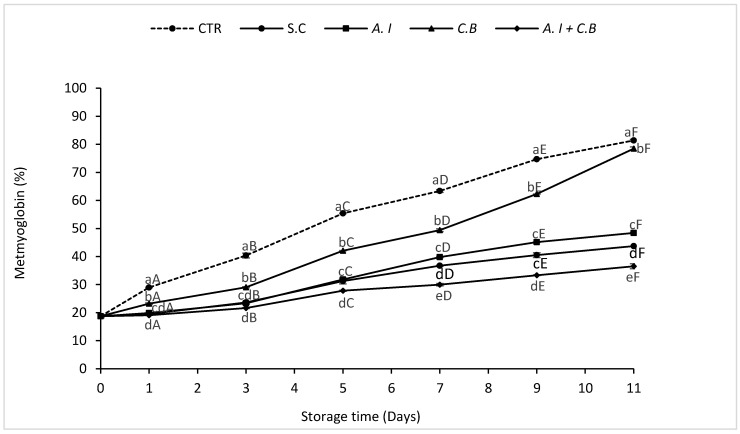
Effects of powdered *A. indica* leaves added at 0.7% (w/w) on MetMb changes in beef patties during 11 days of refrigerated storage at 4 ± 1 °C. CTR (Control sample without antioxidant), S.C (treatment with 0.7% (w/w) synthetic conservative), *A.I* (treatment with 0.7% (w/w) powdered *A. indica* leaves), *C.B* (treatment with 0.7% (w/w) powdered *C. baccatum* fruits) and *A.I + C.B* (treatment with 0.7% (w/w) powdered *A. indica* leaves and 0.7% (w/w) powdered *C. baccatum* fruits). Results represent the mean of three replicates (*n* = 3) and are expressed as mean value ± SD; different letters in the same day indicate significant differences between samples at *p* < 0.05, different capital letters indicate significant differences between storage days at *p* < 0.05 for the same sample.

Figure 4 presented the changes in the MetMb percentage in beef patties treated during refrigerated storage.

The MetMb increased (*p* < 0.05) progressively in all the beef samples as the treatment time was further prolonged. The CTR sample presented the highest percentage estimated from 18.73% at the initial day of storage to 81.39% at the end of the storage period, while *A.I* + *C.B* beef patties presented the lowest percentage that did not exceed 36.55% after 11 days of chilled storage. Beef samples treated with S.C and *A.I* exhibited almost the same MetMb percentage recorded at 43.69% and 48.42%, respectively, at the end of storage time. However, *C.B* beef samples increased gradually and had higher MetMb percentage ranging from 18.73% to 78.47%.

Different studies reported similar results and suggested that free radicals produced during lipid oxidation may damage the structure of muscle fibers and reduce pigmentation [23,50,51].

Redness (a*) is the most important color parameter of meat and meat products [24]. The fading of red color of beef patties during storage can be explained by the oxidation of myoglobin over time when meat decomposes and MetMb starts to be formed [52]. In addition, the main cause of the color change in meat is the oxidation of myoglobin from Fe(II) of myoglobin to Fe(III) giving met-myoglobin (MetMb) [40].

To conclude, the measurement of the beef patties color did not show results in agreement with those determined by TBARS, pH and AOC assays. This may be due to the color change that occurred when the beef patties were mixed with the powdered plants, especially *A. indica* whose leaves darkened the beef patties, hence the red color of raw meat had been masked. However, the determination of MetMb percentage in beef patties was effective to support the color measurements results obtained. 

#### 3.3.4. Hexanal Content

**Figure 5:**
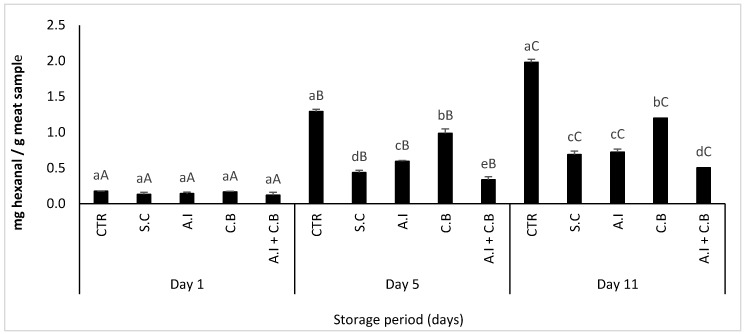
Hexanal content in beef patties during 11 days of refrigerated storage at 4 ± 1 °C. CTR (Control sample without antioxidant), S.C (treatment with 0.7% (w/w) synthetic conservative), *A.I* (treatment with 0.7% (w/w) powdered *A. indica* leaves), *C.B* (treatment with 0.7% (w/w) powdered *C. baccatum* fruits) and *A.I + C.B* (treatment with 0.7% (w/w) powdered *A. indica* leaves and 0.7% (w/w) powdered *C. baccatum* fruits). Results represent the mean of three replicates (*n* = 3) and are expressed as mean value ± SD; different letters in the same day indicate significant difference between samples at *p* < 0.05, different capital letters indicate significant difference between storage days at *p* < 0.05 for the same sample.

The hexanal content of meat samples stored at 4 ± 1 °C was determined at day 1, 5 and 11 and the results obtained are represented in Figure 5.

The hexanal content of meat samples increased significantly over storage time. The first day of storage there were no significant differences observed between S.C and *A.I* beef meat samples, whereas CTR, *C.B* and *A.I + C.B* samples showed significant differences. After 5 days of chilled storage, the content of hexanal increased significantly in all the samples. The highest content of hexanal was observed in CTR and *C.B* samples with 1.29 and 0.99 mg hexanal/g meat sample, respectively, while the lowest hexanal content was observed in *A.I + C.B* sample with 0.34 mg hexanal/g meat sample. The S.C and *A.I* meat samples exhibited almost the same effect and presented hexanal contents estimated at 0.436 and 0.594 mg hexanal/g meat sample, respectively. At the end of the storage period, CTR presented the highest content of hexanal at 1.98 mg hexanal/g meat sample while the *A.I + C.B* sample presented the lowest hexanal content estimated at 0.50 mg hexanal/g meat sample. 

Just like color, aroma is an important criterion that influences the decision of customers to buy meat and meat products [53]. Oxidation reactions cause the creation of volatile compounds. The analysis of these volatile compounds is a good indicator of the oxidation state of the meat products [54]. The main compounds sought is hexanal, which is predominant in the volatile fractions of meat products [24].

Similar observations have also been made by Juntachote et al. [55] about holy basil and galangal in pork meat. Gallego et al. [24] also reported similar results about *Caesalpinia decapetala* showing that natural antioxidants exhibited better antioxidant effect than that shown by synthetic conservatives, when assessed by hexanal formation. 

#### 3.3.5. Antimicrobial Analysis

Presence of colony-forming units evaluated in the control and treated beef samples at the first; fifth and last day of incubation is presented in Table 4.

The number of mesophilic bacteria present in all samples at the first day of incubation was less than 10^4^ CFU/g sample. After 5 days of incubation, number of mesophilic bacteria present in CTR beef meat increased to 4.2 × 10^4^ CFU/g, while the rest of samples kept their effective antibacterial properties. At the last day of the experiment, the antimicrobial activity of *C. baccatum* meat sample became weak, hence the increase in the number of bacteria. Our findings are consistent with previous reports in which bioactive compounds from plants were successfully used to disinfect meat samples [27,46,56].

#### 3.3.6. Sensory Analysis

**Figure 6:**
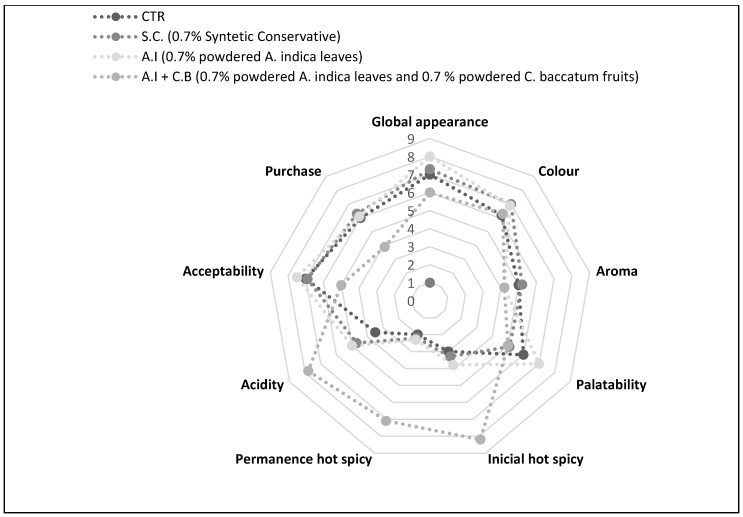
Sensorial analysis of CTR (Control sample without antioxidant), S.C (treatment with 0.7% (w/w) synthetic conservative), *A.I* (treatment with 0.7% (w/w) powdered *A. indica* leaves), and *A.I + C.B* (treatment with 0.7% (w/w) powdered *A. indica* leaves and 0.7% (w/w) powdered *C. baccatum* fruits).

In order to know the total acceptability of the different meat samples, a grade sensorial analysis was made. The results are shown in Figure 6. It should be noted that there are no major differences between the samples, except for the one that incorporates *C. baccatum* fruits that is perceived as extremely spicy. In this sense it depends on the taste and what the consumer is looking for in the hamburger. Its incorporation may be positive, but not always.

## 4. Conclusions

Antioxidant effect of *A. indica* powdered leaves added directly to raw beef patties during refrigerated storage at 4 ± 1 °C was investigated in this study. The results obtained from the analysis of lipid oxidation, changes in pH and color, microbial growth, MetMb formation, hexanal content and antioxidant capacity proved that *A. indica* contains natural antioxidants that might substitute synthetic ones since they presented similar protective effect against deterioration. 

To conclude, the use of *A. indica* as a natural antioxidant in beef meat products might be a good strategy to improve the nutritional value of meat products while ensuring consumers’ safety.

## Figures and Tables

**Table 1:** Total polyphenol content (TPC) and radical scavenging activity (RSA) of *A. indica* leaves and *C. baccatum* fruits aqueous extracts.

Samples	Extract Solvents	TPC (mg GAE/g DW)	RSA: DPPH Assay (mM TE/g DW)
***A. indica***	80% EtOH	47.47 ± 0.03 ^c^	0.37 ± 0.013 ^b^
	80% MeOH	107.41 ± 0.03 ^b^	0.72 ± 0.004 ^a^
	Deionized water	20.93 ± 0.08 ^a^	0.27 ± 0.002 ^c^
***C. baccatum***	80% EtOH	34.78 ± 0.03 ^a^	0.29 ± 0.002 ^b^
	80% MeOH	53.91 ± 0.02 ^b^	0.42 ± 0.001 ^a^
	Deionized water	20.23 ± 0.01 ^c^	0.17 ± 0.004 ^c^

Results represent the mean of three replicates (*n* = 3) and are expressed as mean value ± SD. For each sample different letters in the same column indicate significant differences in each column at *p* < 0.05. DPPH (Diphenyl-1-picrylhydrazyl), MeOH (methanol), GAE (gallic acid equivalent) and TE (Trolox equivalent).

**Table 2:** Antibacterial activity of *A. indica* and *C. baccatum* extracts determined by inhibitory zone assay.

			Inhibitory Zone (mm)			
	Microorganisms	Strains	*A. indica*	*C. baccatum*	*penicillin*	80% MeOH
**Gram+**	*S. aureus*	ATCC 25423	19	22	24	-
	*M. luteus*	ATCC 4698	12	10	17	-
	*Listeria*	ATCC 15313	-	10	15	-
	*B. cereus*	ATCC 11778	-	-	10	-
**Gram−**	*S. paratyphi*	ATCC 9150	10	-	30	-
	*E. coli*	ATCC 25022	21	-	27	-

*A. indica* 80% MeOH extract. *C. baccatum* 80% MeOH extract. Penicillin is used as positive control. Sterile 80% MeOH is used as negative control. Gram+: Gram positive bacteria. Gram−: Gram negative bacteria. No inhibition zone is indicated by (−).

**Table 3:** Color changes (a*, b*, L*) in treated raw beef patties during refrigerated storage at 4 ± 1 °C.

Trait	Day	CTR	S.C	*A.I*	*C.B*	*A.I + C.B*
Redness (a*)	1	39.27 ± 2.37 ^aA^	50.12 ± 0.77 ^bA^	42.65 ± 2.16 ^cA^	48.36 ± 0.37 ^dA^	45.19 ± 1.26 ^cA^
	2	33.32 ± 0.37 ^aB^	49.14 ± 1.32 ^bB^	39.43 ± 0.96 ^cB^	47.02 ± 0.05 ^bB^	38.77 ± 2.14 ^cB^
	3	30.15 ± 0.26 ^aC^	48.89 ± 1.52 ^bB^	38.73 ± 1.03 ^cC^	45.07 ± 0.33 ^dC^	38.23 ± 2.45 ^cB^
	4	27.65 ± 0.71 ^aD^	48.62 ± 0.51 ^bB^	38.31 ± 0.65 ^cC^	43.47 ± 0.23 ^dD^	37.45 ± 1.45 ^cC^
	5	27.12 ± 0.31 ^aD^	45.56 ± 0.21 ^bC^	38.04 ± 0.27 ^cC^	42.11 ± 0.15 ^bE^	37.01 ± 1.32 ^cC^
	7	25.88 ± 0.17 ^aE^	41.96 ± 0.23 ^bD^	36.01 ± 0.25 ^cD^	37.85 ± 1.22 ^cF^	36.04 ± 1.23 ^cD^
	8	25.24 ± 0.12 ^aE^	39.78 ± 1.02 ^bE^	34.29 ± 0.63 ^cE^	31.11 ± 0.47 ^dG^	35.77 ± 1.09 ^cE^
	9	23.99 ± 0,13 ^aF^	39.02 ± 1.01 ^bE^	31.12 ± 0.16 ^cF^	29.63 ± 0.23 ^dH^	35.19 ± 1.47 ^dE^
	10	23.06 ± 0.19 ^aF^	38.45 ± 0.98 ^bF^	28.29 ± 0.77 ^cG^	27.03 ± 0.11 ^cI^	30.44 ± 2.52 ^dF^
	11	22.89 ± 1.23 ^aG^	37.21 ± 1.00 ^bG^	26.64 ± 1.26 ^cH^	25.24 ± 1.14 ^cJ^	30.01 ± 0.99 ^dF^
Yellowness (b*)	1	11.28 ± 0.11 ^aA^	15.25 ± 0.11 ^bA^	14.14 ± 0.67 ^bA^	10.55 ± 1.21 ^dA^	12.25 ± 1.01 ^cA^
	2	10.56 ± 1.32 ^aB^	14.43 ± 1.14 ^bB^	13.49 ± 1.26 ^bB^	9.83 ± 2.01 ^dB^	12.04 ± 1.61 ^cA^
	3	9.89 ± 1.22 ^aC^	13.98 ± 1.22 ^bC^	13.32 ± 1.25 ^bB^	9.78 ± 0.55 ^aB^	11.78 ± 1.44 ^cB^
	4	9.54 ± 1.03 ^aC^	13.43 ± 0.11 ^bC^	12.87 ± 0.42 ^cC^	8.66 ± 1.06 ^dC^	11.59 ± 1.25 ^dB^
	5	9.43 ± 0.15 ^aC^	13.25 ± 0.25 ^bC^	12.44 ± 0.17 ^cC^	8.51 ± 0.09 ^dC^	11.23 ± 1.09 ^eB^
	7	7.45 ± 0.18 ^aD^	12.12 ± 0.13 ^bD^	11.01 ± 0.44 ^cD^	7.88 ± 0.15 ^aD^	10.76 ± 1.21 ^dC^
	8	5.33 ± 0.07 ^aE^	11.69 ± 1.09 ^bE^	10.82 ± 0.73 ^cE^	7.06 ± 0.45 ^dD^	10.66 ± 1.56 ^cC^
	9	5.24 ± 0.04 ^aE^	11.23 ± 0.33 ^bE^	10.12 ± 0.01 ^cE^	6.59 ± 0.16 ^dE^	10.09 ± 1.33 ^cC^
	10	5.17 ± 1.22 ^aE^	10.76 ± 0.26 ^bF^	9.72 ± 0.59 ^cF^	6.13 ± 0.19 ^dE^	9.44 ± 2.47 ^cD^
	11	5.02 ± 1.18 ^aE^	10.27 ± 0.11 ^bF^	9.42 ± 0.19 ^cF^	5.32 ± 1.69 ^aF^	9.21 ± 2.89 ^cD^
Lightness (L*)	1	57.37 ± 0.74 ^aA^	70,79 ± 2.03 ^bA^	68,70 ± 2,24 ^cA^	65.16 ± 2.00 ^dA^	66.15 ± 2.20 ^dA^
	2	56.32 ± 2.17 ^aB^	67.09 ± 3.02 ^bB^	66.46 ± 1,42 ^cB^	65.17 ± 2.02 ^dA^	66.01 ± 2.09 ^cA^
	3	56.01 ± 1.85 ^aB^	65.33 ± 1.23 ^bC^	64.09 ± 1.74 ^cC^	63.46 ± 1.46 ^dB^	65.19 ± 2.33 ^bB^
	4	55.84 ± 1.40 ^aC^	64.70 ± 3.53 ^bC^	62,61 ± 0,33 ^bD^	61.43 ± 1.52 ^cC^	61.23 ± 2.40 ^cC^
	5	54.98 ± 1.78 ^aD^	63.12 ± 2.10 ^bD^	58.12 ± 0.78 ^cE^	59.87 ± 1.96 ^cD^	54.54 ± 2.10 ^aD^
	7	53.13 ± 1.45 ^aE^	60.15 ± 1.64 ^bE^	56.45 ± 1.77 ^cF^	56.16 ± 1.69 ^cE^	52.33 ± 2.44 ^dE^
	8	52.41 ± 0.50 ^aF^	59.23 ± 0.23 ^bF^	54.15 ± 1.26 ^cG^	55.14 ± 1.62 ^cF^	51.46 ± 2.30 ^dF^
	9	51. 26 ± 1.45 ^aG^	56.54 ± 0.19 ^bG^	52. 49 ± 1.65 ^cH^	53.75 ± 1.36 ^dG^	50.73 ± 2.96 ^eG^
	10	47.46 ± 1.85 ^aH^	54.16 ± 0.15 ^bH^	50.46 ± 1.87 ^cI^	51.36 ± 1.64 ^dH^	49.16 ± 2.23 ^eH^
	11	43.23 ± 2.96 ^aI^	54.41± 2.65 ^bF^	49.22± 0.04 ^cJ^	48.04 ± 0.21 ^cI^	47.36 ± 2.08 ^dI^

Results represent the mean of three replicates (*n* = 3) and are expressed as mean value ± SD. CTR (Control sample without antioxidant), S.C (treatment with 0.7% (w/w) synthetic conservative), *A.I* (treatment with 0.7% (w/w) powdered *A. indica* leaves), *C.B* (treatment with 0.7% (w/w) powdered *C. baccatum* fruits) and *A.I + C.B* (treatment with 0.7% (w/w) powdered *A. indica* leaves and 0.7% (w/w) powdered *C. baccatum* fruits). Different letters in the same day indicate significant differences between samples at *p* < 0.05, different capital letters indicate significant differences between storage days at *p* < 0.05 for the same sample.

**Table 4:** Effect of *A. indica* and *C. baccatum* on microbial quality of raw beef meat during refrigerated (4 ± 1 °C) storage.

Aerobic Mesophilic Bacteria Presence	Refrigerated Storage Period (Days)		
	0	5	11
**CTR**	−	+	+
**S.C**	−	−	−
***A.I***	−	−	−
***C.B***	−	−	+
***A.I + C.B***	−	−	−

(−) indicates less of 10^4^ CFU/g sample of aerobic mesophilic bacteria in the meat sample; (+) indicates a number of aerobic mesophilic bacteria between 10^4^ and 10^5^ CFU/g.
